# Clonality-Climate Relationships along Latitudinal Gradient across China: Adaptation of Clonality to Environments

**DOI:** 10.1371/journal.pone.0094009

**Published:** 2014-04-07

**Authors:** Duo Ye, Yukun Hu, Minghua Song, Xu Pan, Xiufang Xie, Guofang Liu, Xuehua Ye, Ming Dong

**Affiliations:** 1 State Key Laboratory of Vegetation and Environmental Change, Institute of Botany, Chinese Academy of Sciences, Beijing, China; 2 Key Laboratory of Hangzhou City for Ecosystem Protection and Restoration, College of Life and Environmental Sciences, Hangzhou Normal University, Hangzhou, Zhejiang, China; 3 Key Laboratory of Ecosystem Network Observation and Modeling, Institute of Geographic Sciences and Natural Resources Research, Chinese Academy of Sciences, Beijing, China; 4 University of Chinese Academy of Sciences, Beijing, China; Institute of Botany, Czech Academy of Sciences, Czech Republic

## Abstract

Plant clonality, the ability of a plant species to reproduce itself vegetatively through ramets (shoot-root units), occurs in many plant species and is considered to be more frequent in cold or wet environments. However, a deeper understanding on the clonality-climate relationships along large geographic gradients is still scarce. In this study we revealed the clonality-climate relationships along latitudinal gradient of entire China spanning from tropics to temperate zones using clonality data for 4015 vascular plant species in 545 terrestrial communities. Structural equation modeling (SEM) showed that, in general, the preponderance of clonality increased along the latitudinal gradient towards cold, dry or very wet environments. However, the distribution of clonality in China was significantly but only weakly correlated with latitude and four climatic factors (mean annual temperature, temperature seasonality, mean annual precipitation, precipitation seasonality). Clonality of woody and herbaceous species had opposite responses to climatic variables. More precisely, woody clonality showed higher frequency in wet or climatically stable environments, while herbaceous clonality preferred cold, dry or climatically instable environments. Unexplained variation in clonality may be owed to the influences of other environmental conditions and to different clonal strategies and underlying traits adopted by different growth forms and phylogenetic lineages. Therefore, in-depth research in terms of more detailed clonal growth form, phylogeny and additional environmental variables are encouraged to further understand plant clonality response to climatic and/or edaphic conditions.

## Introduction

Plant clonality is characterized by the ability of a plant species to reproduce itself vegetatively through ramets (shoot-root units) that are connected by spacers [1]–[3]. It is one of the key life-history strategies for adapting to environmental challenges in many species [4], [5]. Clonal growth enables plants to spread horizontally for exploiting limited resources in patchy environments, to share resource among ramets, and to store resource [1], [6]–[8]. Therefore, clonality has important implications for species survival and population persistence. Clonal plants dominate in many vegetations [5], [9], [10] and their distribution affects the species composition and structure of communities [11]–[14].

Distribution patterns of clonal species strongly depend on environmental conditions. Clonality is often considered to be important for the persistence of plants and the maintenance of plant communities in unfavorable environments, especially in cold or wet habitats [8], [15], [16]. The predominance of clonality is achieved by species turnover of non-clonal species and their replacement by clonal species along temperature and moisture gradients. Previous comparative studies on the flora of central Europe showed that clonal plants were more frequent at lower mean annual temperature (MAT) [5], [8]. Similarly, there is much evidence that clonality is more common in wet habitats, where water-logged conditions can cause oxygen stress [5], [8], [17], [18]. However, the distribution patterns of plant clonality along climatic gradients have been questioned recently [16], [19]. On the one hand, research on the distribution pattern of clonality along the Northeast China Transect (NECT) revealed that clonal plants were more abundant and important in communities at the western part of the NECT, where MAT and mean annual precipitation (MAP) were relatively low [20]. On the other hand, Klimešová and Doležal [19] did not find evidence that a higher proportion of clonal species appeared in cold environments in an analysis of a comprehensive dataset including arctic, alpine and reference regions. Whilst studies on plant clonality-climate relationships along geographic gradients have been accumulating, comprehensive datasets across large spatial gradients are still scarce.

Since clonal plants are considered to be more adapted to stressful (cold, dry or wet) environments than non-clonal plants, here we tested the hypothesis that clonal plants are more frequent in cold or wet habitats. In China, climate varies strikingly and shows clear latitudinal patterns. At the same time vegetation regions in China are very diverse and are broadly distributed from the cold temperate zones to the tropics. We thus expected that: (1) there were significant relationships between plant clonality and annual temperature and/or precipitation, or the seasonality of each of these, across China. However, as these single climatic variables may covary and interact along latitudinal gradients [21], latitude might best explain clonality as an integrative measure of these covarying gradients. Thus we expected that (2) strong latitudinal pattern of plant clonality would be observed over entire China.

## Methods

### Data Collection

**Figure 1:**
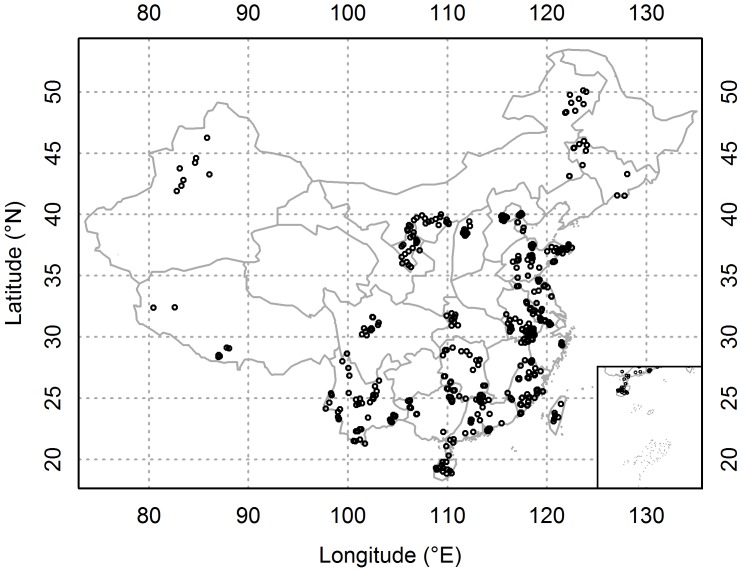
Geographical positions of sampling sites in China.

We collected plant species data based on personal communications (see Acknowledgements) and carried out field investigations in the arid and semi-arid areas of China, authorized by the Chinese Academy of Sciences (CAS). A total of 545 communities across the whole of China, ranging from 18°50'24'' to 50°7'48'' N, and from 80°26'24'' to 128°4'12'' E, were sampled (Figure 1). Floristic surveys using the plot method [22] were carried out by the same methodology. In the forest communities, plant species in the tree, shrub, and herb layers were sampled using one 20×20 m plot, four 2×2 m subplots and four 1×1 m subplots, respectively. In the shrub communities, plants in the shrub and herb layers were sampled using four 2×2 m plots and four 1×1 m subplots, respectively. In the herbaceous communities, plants were sampled using four 1 m×1 m plots. Species richness in each community was determined. Vascular plant species were categorized into non-clonal plants and clonal plants based on simple morphological characters described in the ‘Flora Reipublicae Popularis Sinicae’ [23], or by checking herbarium specimens of species if their descriptions on clonal characteristics were unavailable. We also used the CLO-PLA database [24] as a supplement for plant clonality identification: a species that had any of the 17 clonal growth organs in the CLO-PLA database was considered to be a clonal plant. Except for herbaceous species, woody species comprising trees and shrubs were also included in this study. Clonality of woody species was determined according to the spontaneous clonal growth by layering, rhizome production, or root suckering [25], [26]. The proportion of clonal species in a plant community, representing the relative importance of clonality, was determined by dividing the number of clonal species by the total number of species in a plant community. Totally, there were 4015 species (1360 genera, 227 families, 72 orders) in 545 plots, of which 1579 were determined as clonal species (595 genera, 154 families, 59 orders). Mean annual temperature (MAT, °C), mean annual precipitation (MAP, mm), temperature seasonality (TS) and precipitation seasonality (PS) of each site were extracted from the WorldClim dataset [27] based on its geographical coordinate (Table S1). MAT and MAP represent the annual average trends of heat and moisture for a given site, respectively while temperature seasonality and precipitation seasonality represent intra-annual climate instability.

### Data analysis

Examining the bivariate relationships between variables is an important precursor before using structural equation modeling (SEM) because it allows potentially nonlinear relationships to be identified [28]–[30]. We used both linear and quadratic logistic regressions to test the bivariate relationships between clonality and latitude and each of four climatic variables, respectively, using the glm function with quasibinomial family and logit link in R, because the response variables were overdispersed [31]. In these regressions, we weighted the proportion of clonal species with species richness in each community. Similar procedures were used to uncover the relationship between each climatic factor and clonality for either woody species or herbaceous species due to disparate types of clonality of woody and herbaceous growths (see above). We used the chi-squared test and Nagelkerke's R square to carry out the choice of models on linear or quadratic logistic regressions. These analyses were performed in R package 2.15.0 [32].

We used structural equation modeling (SEM) to quantify the networks of interactions among latitude, climatic variables and clonality. SEM is a modern version of path analysis that allows for both the direct and indirect theoretical causal relationships between networks of intercorrelated variables to be tested [29], [33]. Variables were standardized to similar ranges prior to analysis (latitude and MAT were divided by 10, temperature seasonality by 100, MAP and precipitation seasonality by 1000). The SEMs were fitted using M-plus 6.1 with the binary response variable [34]. An initial SEM was specified based on prior theoretical knowledge. In this model, we assumed that latitude affects climatic variables which in turn affect plant clonality, in which climatic variables may interact. The chi-squared test of model fit was used to determine whether the fit between model and data was adequate (P>0.05).

## Results

### Clonality-climate relationship

**Figure 2:**
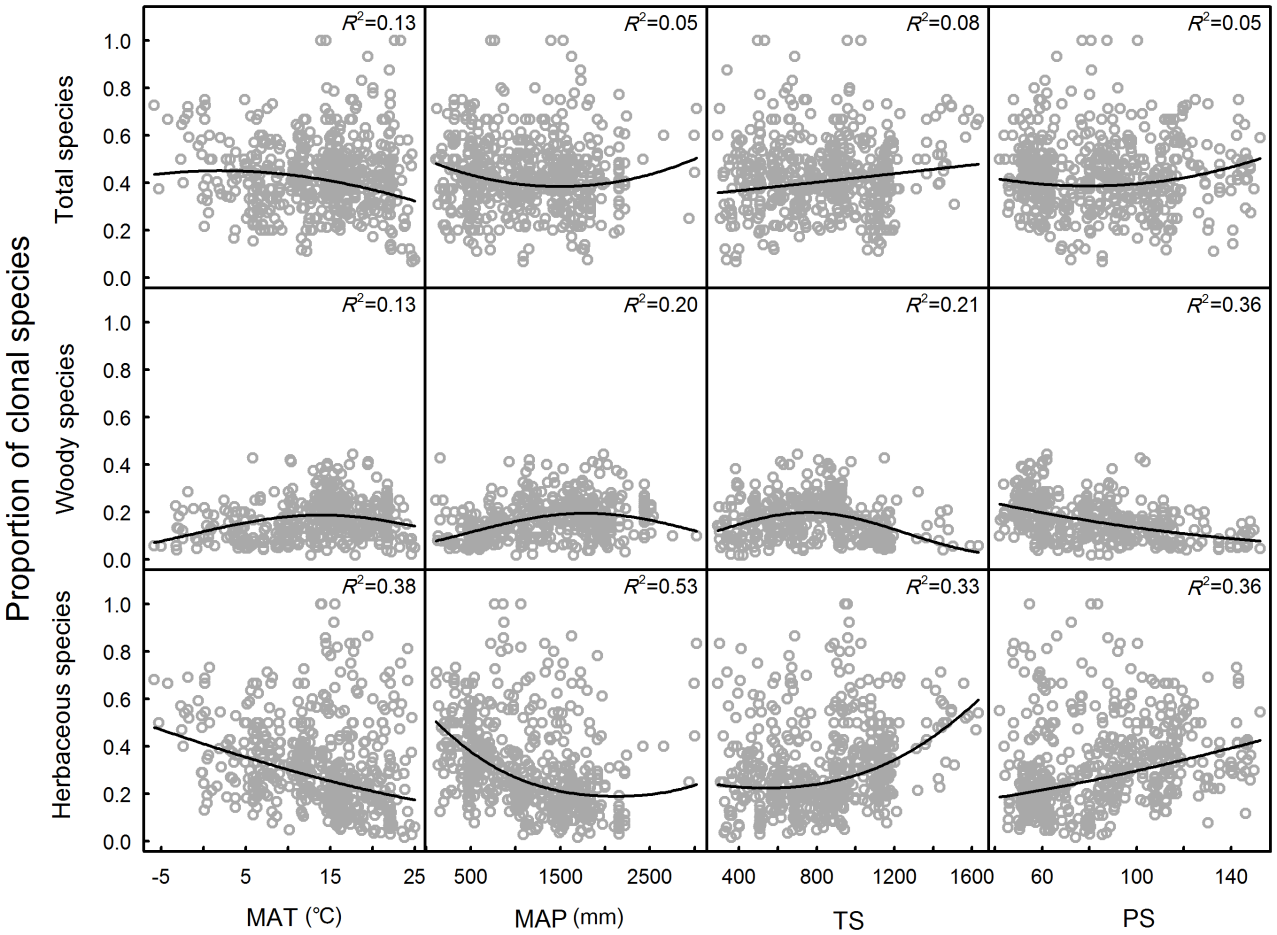
Relationships between proportion of clonal species and climatic variables (MAT: mean annual temperature, MAP: mean annual precipitation, TS: temperature seasonality and PS: precipitation seasonality). a–d: proportion of all clonal species vs climatic variables; e–h: proportion of woody clonal species vs climatic variables; i–l: proportion of herbaceous clonal species vs climatic variables.

Logistic regressions exhibited an increasing trend of the proportion of clonality in plant communities with temperature seasonality (TS) and precipitation seasonality (PS) while it decreased with MAT and MAP (Figure 2a, b, c, d), in which MAT explained 13% of the total variation of clonality. Higher proportions of clonality were observed in the sites with over 2500-mm MAP, but this pattern was only based on few communities. Similar patterns can be seen in the relationships between herbaceous clonality and all climate factors considered (Figure 2i, j, k, l). Woody clonality showed contrasting patterns with total clonality pattern except for TS (Figure 2e, f, g, h), in which woody clonality increased up to a peak and decreased with TS increase. When the proportions of plant clonality were separated based on two contrasting growth forms, the four climate factors could explain much more variation within herbaceous or woody clonality than that in total clonality in a plant community (Figure 2).

### Latitudinal pattern of clonality

**Figure 3:**
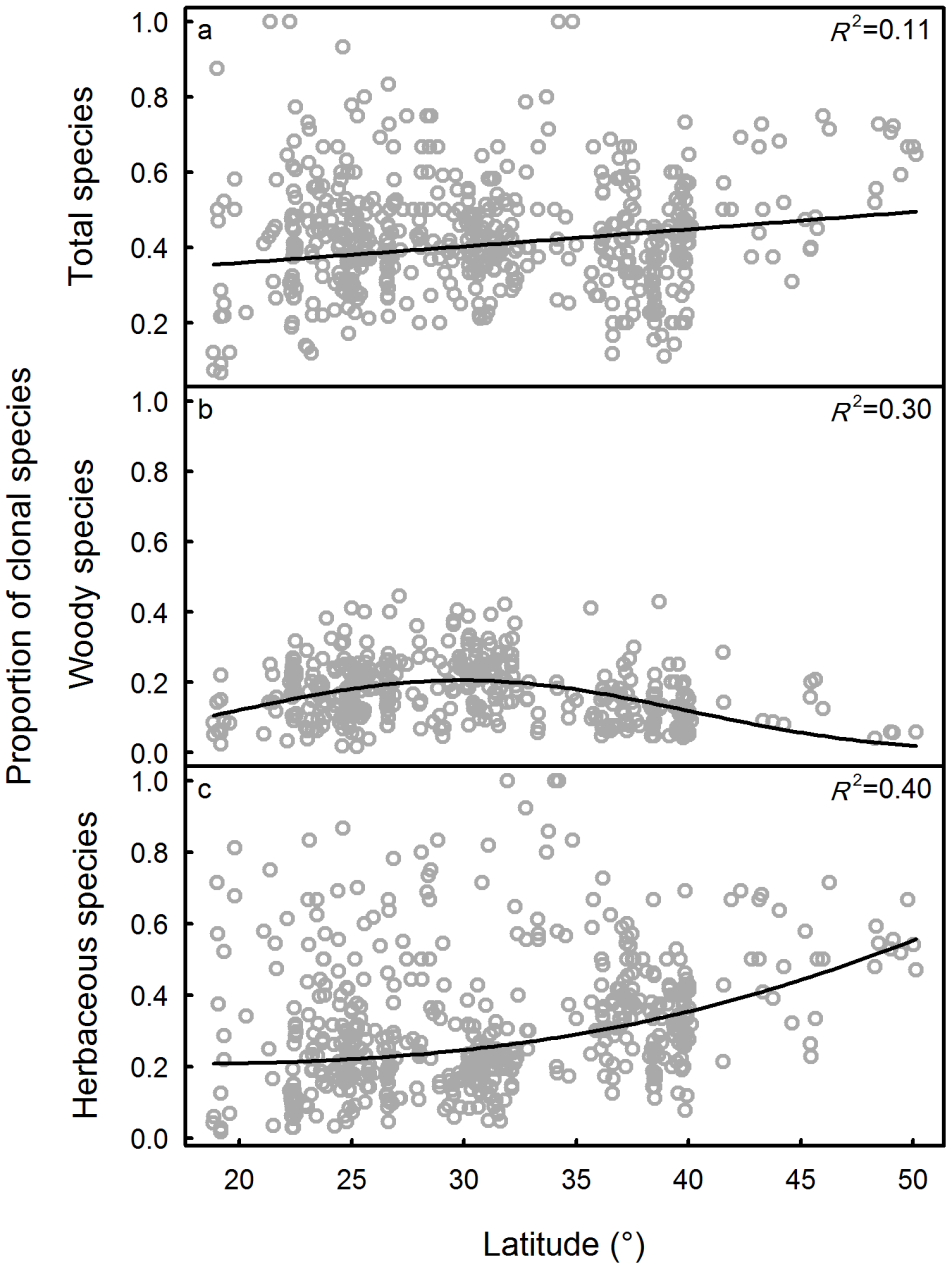
Relationships between proportion of clonal species and latitude. a: all species; b: within woody species; c: within herbaceous species.

There was a positive linear relationship between clonality as a proportion of all species in a community and latitude, since the quadratic model was not significantly better than the linear model (P = 0.86, Figure 3a). Clonality of woody species increased first and then decreased with increasing latitude (Figure 3b). Clonality of herbaceous species increased more steeply with increasing latitude (Figure 3c).

### Structural Equation Modeling

**Figure 4:**
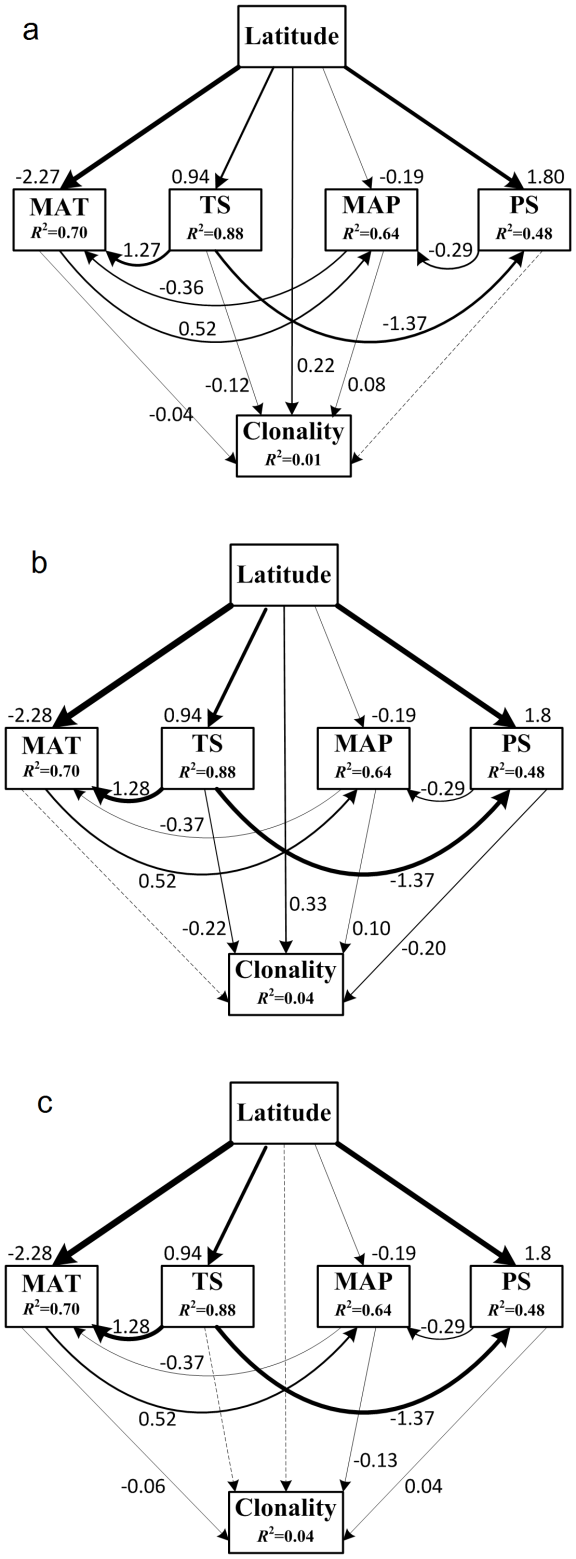
Structural equation models for variation of clonality. Non-significant paths are indicated by dotted arrows. The thickness of the solid arrows reflects the magnitude of the standardized SEM coefficients. Standardized coefficients are listed beside each significant path, as is the variance explained (*R* ^2^) for each endogenous variable. a: all species; b: within woody species; c: within herbaceous species.

The fits between the modified structural equation models and data were adequate for proportion clonality of all species in a community, woody species and herbaceous species (χ^2^_1_ = 0.04, P = 0.83; χ^2^_1_<0.001, P = 0.99; χ^2^_1_<0.001, P = 0.99; respectively). Climate factors and latitude together explained 1, 4 and 4% of the variation in all species (Figure 4a), woody species (Figure 4b) and herbaceous species (Figure 4c), respectively. Latitude had a direct positive effect on variation in proportion clonality of all species but MAP had a negative effect (Figure. 4a). For woody clonality, latitude and MAP had direct positive effects, while TS and PS had negative effects (Figure 4b); for herbaceous clonality, MAT and MAP had negative effects but PS had a negative effect (Figure 4c).

## Discussion

### Latitudinal pattern in clonality

Clonality was expected to increase along the latitudinal gradient. Relatively higher latitude presents more stressful conditions with lower temperature and precipitation and higher seasonality. Clonality was considered be more frequent in these cold or dry conditions. This study provides new, large scale evidence that clonal plants show a latitudinal pattern and increase along a latitudinal gradient across entire China spanning from the tropics to the temperate zones. Latitudinal patterns were better explained by the underlying environmental factors [28], [29]. In previous studies, smaller seeds, shorter plant heights and predominance of herbaceous growth forms at high latitude could be related to low temperature and precipitation [21], [30], [35]. These relationships suggest that the latitudinal gradients of temperature, precipitation and their seasonality in our study may also be the causes of the distribution pattern of plant clonality.

Clonality of woody species mainly decreased with latitude but the trend was reversed in herbaceous species. This suggests that different growth forms may have different adaptive clonal strategies to the complex environmental conditions. As a matter of fact, herbaceous clonal plants dominate the latitudinal pattern of clonality owing to their species number being almost twice as that of woody clonal plants in the communities sampled. Temperature, precipitation and their seasonality therefore are direct forcing drivers of the latitudinal pattern of clonality.

### Adaptation of clonality on climate

In the present study, we found that clonality increased with decreasing temperature and with increasing precipitation. Thus, this study provides support for the hypothesis that clonal plants are more frequent in cold or very wet habitats across a huge climatic gradient [5], [8].

However, clonality was significantly but only weakly correlated with the climatic factors. The lack of strong correlation may be due to issues of growth form. We found that clonality of woody species and herbaceous species had opposite response to climatic factors. Woody clonality was more frequent in very wet or climatically stable circumstances, while herbaceous clonality was more frequent in cold, dry or climatically instable conditions. Several other studies have found that different clonal traits underpinned different adaptative strategies with respect to environmental factors [36]–[38]. This suggests that different responses to environmental gradients may actually be due to different advantages brought by different clonal growth forms or organs.

Increasing precipitation and decreasing climatic seasonality represent the favourable climates associated with stronger competition. Clonality gives woody plants more vigour to compete in the increasingly competitive habitats along these gradients [39], [40]. Cold temperatures exert abiotic limitations on sexual reproduction, especially in herbaceous plants, such as limiting the capacity either to flower or to fertilize ovules [41], inhibition of fruit or seed development [42], seed germination [43] and seedling establishment [44]. However, horizontal spreading of clonal genets can enable herbaceous plants to migrate over short distances and to occupy space under conditions that limit seedling recruitment including poor seed production and dispersal [4]. Belowground physiological integration of rhizomes and roots [45], [46] and resource storage in organs such as tubers and bulbs [5], [46], [47], could enable the herbaceous clonal plants to overcome drought periods [48], [49] and provides them with a long lifespan and asexual reproduction insurance.

## Conclusions

Our results are consistent with the hypothesis that clonality is more frequent in cold, dry or particularly wet environments. Clonality in China adheres to a latitudinal pattern and increases with increasing latitude. However, the distribution of clonality in China is significantly but only weakly correlated with latitude and climatic factors. This may be due to clonality of woody and herbaceous species adopting opposite response strategies with respect to climatic variables. More precisely, woody clonality shows higher frequency in wet or climatically stable circumstances, while herbaceous clonality prevails in cold, dry or climatically unstable conditions. It is also possible that, across or within growth forms, stronger climatic patterns might be seen within certain phylogenetic lineages and that different patterns for different lineages cancel each other out in the complete species set. Further unexplained variation in clonality may be owed to the influences of other environmental conditions not taken into account here, including edaphic variation. Therefore, in-depth research in terms of more detailed clonal growth forms or phylogenetic lineages and additional more environmental variables are encouraged to further enhance our understanding of plant clonality response to climatic and/or edaphic gradients.

## Supporting Information

Table S1 Coordinates of sites and their species richness and proportion of clonal plants.
(XLS)
