# Correction: Clonality-Climate Relationships along Latitudinal Gradient across China: Adaptation of Clonality to Environments

**DOI:** 10.1371/journal.pone.0103175

**Published:** 2014-07-15

**Authors:** The PLOS ONE Staff

Figure 2 is missing subplot labels that correspond to the figure legend. The author has provided a corrected copy here.

**Figure 2:**
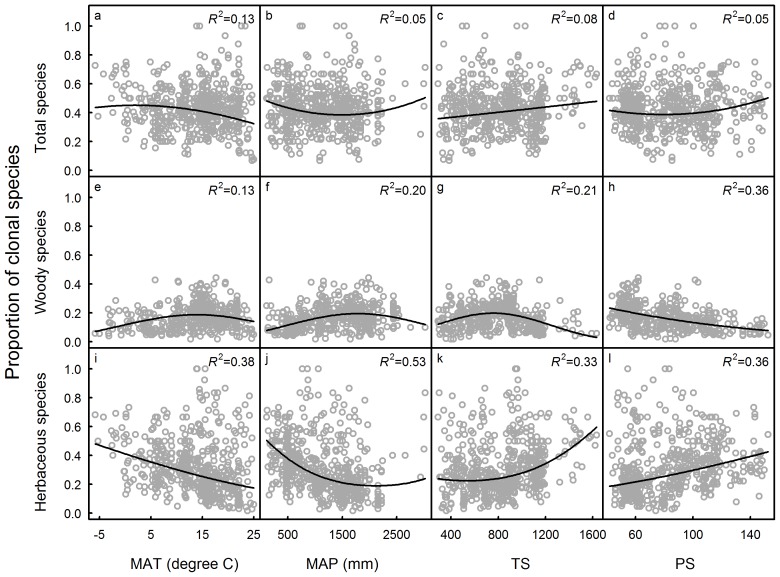
Relationships between proportion of clonal species and climatic variables (MAT: mean annual temperature, MAP: mean annual precipitation, TS: temperature seasonality and PS: precipitation seasonality). a–d: proportion of all clonal species vs climatic variables; e–h: proportion of woody clonal species vs climatic variables; i–l: proportion of herbaceous clonal species vs climatic variables.
